# Sodium buffered formic acid concentration and feed pH is stable over a 3-month period

**DOI:** 10.1093/tas/txab085

**Published:** 2021-05-13

**Authors:** A R Huss, C K Jones, C R Stark, S A Fleming, R N Dilger, J A Jendza

**Affiliations:** 1 Department of Grain Science & Industry, Kansas State University, Manhattan, KS 66506, USA; 2 Department of Animal Sciences & Industry, Kansas State University, Manhattan, KS 66506, USA; 3 Traverse Science, Champaign, IL 61820, USA; 4 Department of Animal Sciences, University of Illinois at Urbana-Champaign, Urbana, IL 61801, USA; 5 BASF, Florham Park, NJ 07932, USA

**Keywords:** broiler, formic acid, feed, hygiene, pelleting, swine

## Abstract

Promoting feed hygiene with organic acids is an effective method to prevent foodborne illnesses from bacterial infection. The stability and acidification of mash and pelleted feed with sodium buffered formic acid was investigated. The acid product was incorporated to reach total formate inclusion levels of 0, 6, or 12 g/kg for swine nursery feed; 0, 4, or 9 g/kg for swine finishing feed; and 0, 3, or 6 g/kg for broiler grower feed. Samples were analyzed for total formate and pH on d 4, 32, 60, or 88 post-manufacturing. The concentration of formate remained stable across an 88-d period (*P* < 0.01). Treatment with the formic acid product decreased feed pH with increasing inclusion levels (all *P* < 0.01). Within each inclusion level of acid and across time, pH tended to increase in pelleted feed and decrease in mash feeds (all *P* < 0.01); however, these changes were small (0.1 units pH). These data suggest that sodium buffered formic acid can be applied to both mash and pelleted feed to provide continuous acidification over a 3-month period.

## INTRODUCTION

In the United States, an estimated 3,645,773 people acquired foodborne infections from bacterial infection in 2006, with nearly a third coming from *Salmonella* spp. nontyphoidal alone (Scallan et al., 2011). With an estimated hospitalization rate of 27.2% and 378 annual deaths, strategies to combat *Salmonella* infections are warranted (Scallan et al., 2011). Reported isolates of *Salmonella* serotype Enteritidis were greatest in infants and children 0–4 years of age exhibit from 1968 to 2012, and the source of non-human isolates were greatest from chicken (50–80% of isolates from chicken) (CDC 2013). Considering the prevalence and impact of this foodborne pathogen, implementing strategies to mitigate the presence of *Salmonella* at the animal level provides a practical solution to the problem.

As reviewed by Jones (2011), control of *Salmonella* in animal feed includes the prevention of contamination, reduction of multiplication, and procedures for killing pathogens. The use of organic acids in feed can act at the last step to kill *Salmonella* and prevent future re-contamination, whether alone or in combination with thermal processing via pelleting (Jones 2011). Pelleting alone may be insufficient to completely kill and/or prevent the re-contamination of pellets, hence pelleting along with organic acid treatment represents an effective measure to promote feed hygiene (Hinton and Linton 1988).

In general, organic acids in feed have been demonstrated to reduce the pH of digesta, improve protein and energy digestibility of feed, and exhibit antimicrobial activity (Dibner and Buttin 2002). Regarding antimicrobial mechanisms, at low pH undissociated organic acids may diffuse into the cytoplasm of bacteria where they dissociate and reduce intracellular pH, thereby disrupting pH sensitive enzymatic processes (Suiryanrayna and Ramana 2015), and leading to both a bacteriostatic and bactericidal effect. A host of organic acids have been used as feed hygiene agents, with formic and propionic acid being relatively well studied (Dittoe et al., 2018). A 2015 report by the European Food and Safety Administration (EFSA) Panel on Additives and Products or Substances used in Animal Feed (FEEDAP) on the safety of formic acid and its sodium salt identified no adverse effects of their use as feed hygiene agents in complete feed for pigs (up to 12 g/kg) and other animal species (up to 10 g/kg), and is safe for consumers (EFSA FEEDAP Panel, 2015). In 2019, sodium formate was deemed potentially efficacious as a hygiene condition enhancer after review of submitted in vitro data demonstrating its ability to reduce microbial contamination of feed (EFSA FEEDAP Panel, 2019). In light of the known functions of formic acid and formate salts, the following research was conducted to assess the stability and acidification of sodium buffered formic acid (Amasil NA, BASF Corporation, Ludwigshafen, Germany) in three types of feed (swine nursery, swine finisher, and broiler grower) as compared with pelleting.

## MATERIALS AND METHODS

No live-animal work was performed as part of this research; thus no Institutional Animal Care and Use Committee approval was required.

### Feed Manufacture

One batch of mash feed was manufactured at North Carolina State University for each of three species-appropriate corn and soybean meal-based formulations (**Table 1**) tested [1) swine nursery feed, 2) swine finishing feed, and 3) broiler grower feed] for a total of 6,123 kg of base feed. Each batch of feed was subsequently divided into three lots to permit inclusion levels of formate. Per inclusion level, the sodium buffered formic acid product (61% formic acid, 20.5% sodium formate, and 18.5% water) was incorporated into three nonconsecutive (to avoid contamination from the previous lot) feed lots via the mixer to attain total formate inclusion concentrations of 0, 6, or 12 g/kg for swine nursery feed; 0, 4, or 9 g/kg for swine finishing feed; and 0, 3, or 6 g/kg for broiler grower feed. Each lot was then further divided by form and either pelleted or left in mash form. Ten representative samples from each lot of treatment feed (mash form) were obtained from a variety of locations within the mixer with a feed probe using established methods (Association of American Feed Control Officials 2000). After mash feed samples were collected, the remaining feed lots were pelleted at 82.2 °C (180 °F) and cooled for 15 minutes in a batch cooler, at which point 10 representative samples of pelleted feed were collected from each lot of treatment feed (pelleted form). All samples were shipped to Kansas State University and analyzed for formate and pH on d 4, 32, 60, and 88 post-manufacturing, as detailed below.

**Table 1.** Base formulation of the experimental diets

	Swine		
Ingredient, g/kg	Nursery	Finisher	Broiler grower
Corn	589	862	564
Soybean meal, 46.5%	253.5	117	361.5
Select menhaden fish meal	12.5	0	0
Spray-dried whey	100	0	0
Spray-dried blood cells	12.5	0	0
Soy oil	0	0	28.5
Monocalcium P, 21% P	8.5	4.5	17
Limestone	8	9	15
Salt	3	3.5	3
Zinc oxide	2.5	0	0
Swine vitamin premix with phytase^1^	2.5	0.75	0
Swine trace mineral premix^2^	1.5	0.75	0
Poultry vitamin and trace mineral premix^3^	0	0	2.5
Lysine HCl	3.25	2.25	1.5
DL-Methionine	1.5	0	3.15
L-Threonine	1.3	0.35	0.5
Sodium bicarbonate	0	0	2.25
Monensin sodium	0	0	0.55
Choline chloride	0	0	0.6
Total, g	1000	1000	1000

^1^Supplied per kg of feed: 11,023 IU vitamin A, 1,378 IU vitamin D3, 44 IU vitamin E, 4 mg vitamin K, 8 mg riboflavin, 28 mg pantothenic acid, 50 mg niacin, and 0.04 mg vitamin B12.

^2^Supplied per kg of feed: 40 mg Mn from manganese oxide, 17 mg Fe from iron sulfate, 17 mg Zn from zinc sulfate, 2 mg Cu from copper sulfate, 0.30 mg I from calcium iodate, and 0.30 mg Se from sodium selenite.

^3^Supplied per kg of feed: 13,200 IU vitamin A, 4,000 IU vitamin D3, 33 IU vitamin E, 0.02 mg vitamin B12, 0.13 mg biotin, 2 mg menadione (K3), 2 mg thiamine, 6.6 mg riboflavin, 11 mg d-pantothenic acid, 4 mg vitamin B6, 55 mg niacin, 1.1 mg folic acid, and 120 mg manganese, 120 mg zinc, 80 mg iron, 10 mg copper, 2.5 mg iodine, and 1 mg cobalt.

### Sample Analyses

**Figure 1.**
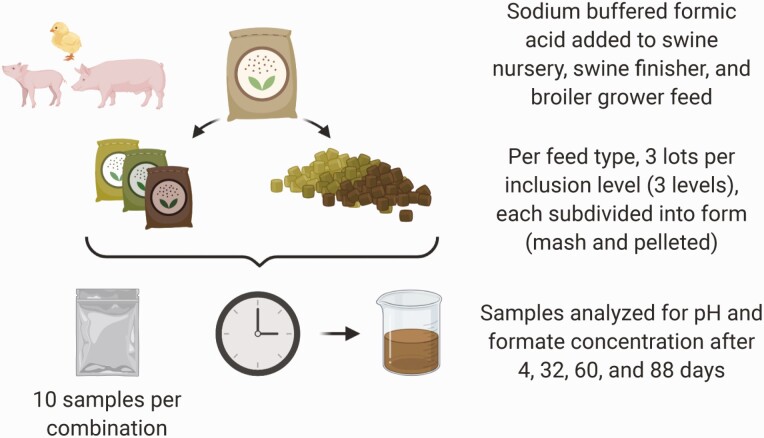
Sodium buffered formic acid was added to three types of feed (poultry, swine nursery, swine finisher, and broiler grower), mashed or pelleted, and samples were analyzed over 88 days to assess pH and formate concentration. Samples were collected across three feed types, three lots of treated mash feed per each of three formate inclusion concentrations (varied by feed type), and two feed forms (mash vs. pelleted), for a total of 54 combinations from which 10 independent subsamples were collected for analysis across four time points.

All samples were collected into sealed, plastic bags (Whirl-Pak; Madison, WI) and stored at room temperature pending analyses. Representative mash and pelleted feed samples were initially processed by milling through a 0.5 mm screen using a Retsch grinder (Model ZM1; Retsch Inc., Newton, PA). To perform both the pH and formate analyses, 10 g of a ground, representative sample was placed in a 600-mL beaker and mixed with 290 mL of distilled, deionized water using a magnetic stir plate (Thermolyne Nuova Dubuque, IA). The beaker was then covered with laboratory film (Parafilm M; Amcor, Oshkosh, WI), chilled at –20 °C for 30 min, stirred, and subsampled. The subsample was then centrifuged at 10,900 × *g* at 4 °C for 60 min and the resulting supernatant was removed via pipette. The pH of the supernatant was measured by pH probe (Accumet Excel XL25, Fisher Scientific, Pittsburgh, PA) and the formate concentration was measured using a commercial colorimetric kit following the manufacturer’s instructions (MAK059-1KT; Sigma-Aldrich, St. Louis, MO). Reported pH and formate values represent the mean of three replicates performed on each of the samples that were collected [three feed types (swine nursery, swine finisher, broiler chicken), three lots of treated mash feed per each of three formate inclusion concentrations (varied by feed type), two feed forms (mash vs. pelleted), for a total of 54 combinations from which 10 independent subsamples were collected for analysis (**Fig. 1**)].

### Statistics

Data were analyzed by regression using the lmer function from the lme4 package (Bates et al., 2015) in R version 3.6.2 (R Core Team 2019). To normalize data, pH was log transformed for analysis and resulting data reflect untransformed values. Two models were used. In the first model, outcome variables (pH and concentration) were regressed against form, inclusion, and their interaction as fixed, between-subject effects with replicate nested within lot as random effects. This model was analyzed sliced by time. In the second model, outcome variables were regressed against time as a repeated, within-subject effect, with replicate nested within lot. This analysis was performed for each level of form by inclusion.

## Results

Across all three feed types (swine nursery, swine finisher, and broiler grower,) the effects observed were similar (**Tables 2–4**). Across all feed types and times sampled, formate concentration did not differ between mash and pelleted feed (fixed effect of form, all *P* ≥ 0.28), concentration increased with inclusion level (fixed effect of inclusion, all *P* < 0.01), and no interactions between form and inclusion were found (all *P* ≥ 0.21). Furthermore, concentration did not change with time (all *P* ≥ 0.05).

**Table 2.** Effects of increasing inclusion levels of sodium buffered formic acid on formate concentration and pH of swine nursery feed^1^

Form:	Mash			Pelleted			Pooled SEM	*P*=		
Inclusion:	0 g/kg	6 g/kg	12 g/kg	0 g/kg	6 g/kg	12 g/kg		Form	Inclusion	Form × Inclusion
Formate concentration, g/kg										
d 4	0.06^c^	6.06^b^	12.09^a^	0.06^c^	6.02^b^	12.03^a^	0.560	0.49	<0.01	0.87
d 32	0.25^c^	6.00^b^	12.02^a^	0.07^c^	6.08^b^	11.96^a^	0.080	0.34	<0.01	0.21
d 60	0.06^c^	6.02^b^	12.05^a^	0.06^c^	6.04^b^	12.02^a^	0.120	0.97	<0.01	0.96
d 88	0.12^c^	5.99^b^	12.05^a^	0.06^c^	6.04^b^	12.02^a^	0.070	0.68	<0.01	0.49
Pooled SEM	0.116	0.157	0.035	0.06	0.07	0.039	-	-	-	-
Time, *P*=	0.33	0.95	0.49	0.99	0.94	0.20	-	-	-	-
pH^2^										
d 4	6.40^a^	5.78^c^	5.30^e^	6.15^b^	5.63^d^	5.08^f^	0.021	<0.01	<0.01	<0.01
d 32	6.04^b^	5.45^d^	4.90^f^	6.52^a^	5.99^c^	5.41^e^	0.024	<0.01	<0.01	<0.01
d 60	6.22^b^	5.64^d^	5.10^f^	6.34^a^	5.81^c^	5.25^e^	0.020	<0.01	<0.01	0.01
d 88	6.20^b^	5.60^d^	5.12^f^	6.33^a^	5.81^c^	5.25^d^	0.021	<0.01	<0.01	<0.01
Pooled SEM	0.007	0.016	0.012	0.006	0.006	0.007	-	-	-	-
Time, *P*=	< 0.01	< 0.01	< 0.01	< 0.01	< 0.01	< 0.01	-	-	-	-

^1^A mash basal diet was manufactured and divided into three treatments of Amasil^®^ NA inclusion concentration (0, 3, or 6 g formate/kg). Three 500-lb lots of each treatment (nine total) were blended and pelleted. Ten samples were collected at the mixer (mash) and after pelleting (pelleted) of each lot and analyzed for stability of formate concentration and pH across days 4, 32, 60, and 88 post-manufacture.

^2^Values were log transformed prior to analysis to normalize distribution. Displayed values are untransformed.

^a–f^Means within a row that do not share a common letter differ *P* < 0.05.

**Table 3.** Effects of increasing inclusion levels of sodium buffered formic acid on formate concentration and pH of swine finishing feed^1^

Form:	Mash			Pelleted			Pooled SEM	*P*=		
Inclusion:	0 g/kg	4 g/kg	9 g/kg	0 g/kg	4 g/kg	9 g/kg		Form	Inclusion	Form × Inclusion
Formate concentration, g/kg										
d 4	0.29^c^	4.12^b^	8.95^a^	0.30^c^	3.97^b^	9.01^a^	0.123	0.69	<0.01	0.37
d 32	0.00^c^	4.06^b^	9.07^a^	0.12^c^	4.08^b^	9.02^a^	0.079	0.49	<0.01	0.32
d 60	0.06^c^	4.12^b^	8.95^a^	0.06^c^	4.02^b^	9.04^a^	0.138	0.99	<0.01	0.67
d 88	0.19^c^	4.10^b^	9.01^a^	0.27^c^	4.02^b^	9.00^a^	0.099	0.99	<0.01	0.48
Pooled SEM	0.085	0.103	0.079	0.100	0.053	0.044	-	-	-	-
Time, *P*=	0.05	0.97	0.67	0.22	0.29	0.96	-	-	-	-
pH^2^										
d 4	6.30^a^	5.79^c^	5.02^e^	6.10^b^	5.54^d^	4.86^f^	0.022	<0.01	<0.01	<0.01
d 32	6.13^b^	5.50^d^	4.73^f^	6.52^a^	5.97^c^	5.35^e^	0.033	<0.01	<0.01	< 0.01
d 60	6.11^b^	5.65^c^	4.88^e^	6.31^a^	5.76^c^	5.11^d^	0.044	<0.01	<0.01	0.18
d 88	6.22^b^	5.65^d^	4.88^f^	6.31^a^	5.76^c^	5.11^e^	0.020	<0.01	<0.01	< 0.01
Pooled SEM	0.050	0.006	0.012	0.040	0.011	0.028	-	-	-	-
Time *P*=	0.11	< 0.01	< 0.01	< 0.01	< 0.01	< 0.01	-	-	-	-

^1^A mash basal diet was manufactured and divided into three treatments of Amasil^®^ NA inclusion concentration (0, 3, or 6 g formate/kg). Three 500-lb lots of each treatment (nine total) were blended and pelleted. Ten samples were collected at the mixer (mash) and after pelleting (pelleted) of each lot and analyzed for stability of formate concentration and pH across days 4, 32, 60, and 88 post-manufacture.

^2^Values were log transformed prior to analysis to normalize distribution. Displayed values are untransformed.

^a–f^Means within a row that do not share a common letter differ *P* < 0.05.

**Table 4.** Effects of increasing inclusion levels of sodium buffered formic acid on formate concentration and pH of broiler grower feed^1^

Form:	Mash			Pelleted			Pooled SEM	*P*=		
Inclusion:	0 g/kg	3 g/kg	6 g/kg	0 g/kg	3 g/kg	6 g/kg		Form	Inclusion	Form × Inclusion
Formate concentration, g/kg										
d 4	0.11^c^	3.05^b^	6.09^a^	0.00^c^	3.05^b^	6.06^a^	0.067	0.31	<0.01	0.65
d 32	0.06^c^	3.13^b^	6.05^a^	0.05^c^	3.00^b^	6.06^a^	0.064	0.31	<0.01	0.35
d 60	0.12^c^	2.97^b^	5.99^a^	0.06^c^	3.05^b^	5.96^a^	0.064	0.90	<0.01	0.51
d 88	0.06^c^	3.15^b^	6.07^a^	0.06^c^	3.02^b^	6.07^a^	0.061	0.28	<0.01	0.37
Pooled SEM	0.071	0.056	0.050	0.049	0.044	0.065	-	-	-	-
Time, *P*=	0.90	0.09	0.36	0.36	0.80	0.56	-	-	-	-
pH^2^										
d 4	6.24^a^	5.90^c^	5.56^f^	6.14^b^	5.84^d^	5.59^e^	0.008	<0.01	<0.01	<0.01
d 32	6.05^c^	5.68^e^	5.40^f^	6.45^a^	6.14^b^	5.82^d^	0.023	<0.01	< 0.01	<0.01
d 60	6.14^ab^	5.80^c^	5.52^d^	6.20^a^	6.00^b^	5.71^c^	0.044	<0.01	<0.01	0.18
d 88	6.144^b^	5.80^d^	5.49^f^	6.30^a^	5.99^c^	5.71^e^	0.010	<0.01	<0.01	< 0.01
Pooled SEM	0.007	0.008	0.016	0.05	0.006	0.006	-	-	-	-
Time, *P*=	< 0.01	< 0.01	< 0.01	0.01	< 0.01	< 0.01	-	-	-	-

^1^A mash basal diet was manufactured and divided into three treatments of Amasil NA inclusion concentration (0, 3, or 6 g formate/kg). Three 500-lb lots of each treatment (nine total) were blended and pelleted. Ten samples were collected at the mixer (mash) and after pelleting (pelleted) of each lot and analyzed for stability of formate concentration and pH across days 4, 32, 60, and 88 post-manufacture.

^2^Values were log transformed prior to analysis to normalize distribution. Displayed values are untransformed.

^a–f^Means within a row that do not share a common letter differ *P* < 0.05.

For all feed types, pH was found to be sensitive across time (fixed effect of time, all *P* < 0.01, for mash feed at the 0 g/kg inclusion level *P* = 0.11). Pelleted samples exhibited increases in pH after 32 days which then returned closer to original levels by day 88, whereas mash samples exhibited the opposite effect. Overall, the changes in pH over time within inclusion level were relatively small. Feed pH was higher in pellets than mash (fixed effect of form, all *P* < 0.01), lower with increasing inclusion levels (fixed effect of inclusion, all *P* < 0.01), and an interaction between form and inclusion was found (all *P* < 0.01, with the exception of d88 for swine finishing and broiler grower feed). Specifically, pH was lowest in mash feed and at higher concentrations of formate.

## Discussion

This research was conducted to assess the stability of feed grade sodium buffered formic acid in feed under practical conditions. We found that total formate was stable in feed over an 88-d period when included at graded concentrations (3–12 g/kg) and exhibited differing effects on feed pH when measured in mash vs. pelleted feeds. For example in broiler grower mash feed, pH was reduced by approximately 0.1 units after 88 days, whereas it exhibited a 0.1 increase in pelleted feed. In both feed forms, pH changed most significantly after 32 days, after which it returned closer to initial levels. While these changes were statistically significant, the small change in pH (0.1) is likely biologically insignificant. By the end of the study, regardless of feed type tested (swine nursery, swine finisher, or broiler grower), pelleted feed exhibited a higher pH than mash feed. However, this too was a small difference in pH (0.23 at most) on day 88.

The reduction in feed pH with addition of organic acids was expected. Organic acids have long been known to reduce the pH of mixed feeds (Barker et al., 1973) and ingredients (Axmann et al., 2017), and this is true in pelleted feeds as well (Partanen et al., 2002). Interestingly, others have also found that while formic acid reduces feed pH initially, measured feed pH may subsequently rise over time, potentially due to the volatility of formic acid (Al-Natour and Alshawabkeh 2005). Regardless, the dose-dependent response of adding a formic acid product to feed is clear, and the change over time was slight or absent entirely.

Clearly, a range of studies have demonstrated the ability of organic acids to reduce feed pH. Given formic acid containing products are cheaper alternatives to the cost of steam conditioning during pelleting for the purposes of decontamination (Hansen et al., 1995), most studies investigated feed ingredients or mash feed, and not pelleted feed. Pertinent to the present study, pelleted feed with 10 g/kg organic acids (formic acid alone or in combination with propionic acid) reduced *Salmonella* by 3.5 or more log_10_ CFU (Koyuncu et al., 2013). Given organic acids can prevent bacterial contamination, using high steam conditioning temperatures for the purposes of de-contaminating feed may be redundant. Using sodium buffered form acid in place of pelleting (or high temperature steam conditioning) might allow feed mill operators to the reduce costs and energy usage of pelleting if pelleting was chosen for its ability to de-contaminate feed. Of course, other considerations for the choice of conditioning temperature should be taken into account, such as its effect on the digestibility of feed.

## CONCLUSION

We assessed the concentration and pH of sodium buffered formic acid over a 3-month period between mash and pelleted forms. The concentration of the product exhibited high stability in mash and pelleted feed, with increasing inclusion levels reducing feed pH, the extent of which varied by form. Furthermore, stability was shown to be consistent across swine nursery, swine finisher, and broiler grower feeds. These data may help producers better understand the use of organic acids in mash and pelleted feed for their given applications.
